# Enhanced Timing Performance of Dual-Ended PET Detectors for Brain Imaging Using Dual-Finishing Crystal Approach

**DOI:** 10.3390/s24206520

**Published:** 2024-10-10

**Authors:** Guen Bae Ko, Dongjin Kwak, Jae Sung Lee

**Affiliations:** Brightonix Imaging Inc., Seoul 04782, Republic of Korea; guenbko@gmail.com (G.B.K.); tiago.kwak@brtnx.com (D.K.)

**Keywords:** positron emission tomography, brain imaging, silicon photomultiplier, time of flight, depth of interaction, dual-ended readout, scintillation crystal

## Abstract

This study presents a novel approach to enhancing the timing performance of dual-ended positron emission tomography (PET) detectors for brain imaging by employing a dual-finishing crystal method. The proposed method integrates both polished and unpolished surfaces within the scintillation crystal block to optimize time-of-flight (TOF) and depth-of-interaction (DOI) resolutions. A dual-finishing detector was constructed using an 8 × 8 LGSO array with a 2 mm pitch, and its performance was compared against fully polished and unpolished crystal blocks. The results indicate that the dual-finishing method significantly improves the timing resolution while maintaining good energy and DOI resolutions. Specifically, the timing resolution achieved with the dual-finishing block was superior, measuring 192.0 ± 12.8 ps, compared to 206.3 ± 9.4 ps and 234.8 ± 17.9 ps for polished and unpolished blocks, respectively. This improvement in timing is crucial for high-performance PET systems, particularly in brain imaging applications where high sensitivity and spatial resolution are paramount.

## 1. Introduction

The demand for high-performance PET detectors has been increasing due to the rising interest in next-generation high-performance PET system such as brain-dedicated PET and long-axial field-of-view (FOV) PET systems [1,2,3,4,5]. Time of flight (TOF) has become an essential technology in PET detectors for these systems because it enhances the image signal-to-noise ratio, improves effective sensitivity, and reduces errors in corrections [6,7,8]. Additionally, the need for depth-of-interaction (DOI) capabilities is growing, as these systems experience more parallax errors compared to conventional whole-body PET. Consequently, there has been active research and development focused on high-performance PET detectors that integrate both TOF and DOI functionalities.

The efforts toward developing TOF/DOI detectors can be categorized into several groups. An approach called the relative offset method involves stacking layers of scintillation crystals with a half-pitch offset [3,9,10]. This method facilitates the easy distinction of DOI within the scintillation crystal map and supports the use of multiplexed readout. However, it has the disadvantage of increased production complexity when attempting to achieve multiple DOI layers. Additionally, the light transmission at the crystal layer interfaces is reduced, leading to optical losses, which can negatively impact the TOF performance. Some approaches leverage differences in light dispersion depending on DOI [11,12,13,14]. These methods allow for DOI discrimination without increasing the number of photosensors, which is advantageous. Typically, differences in light dispersion are achieved by varying the structure of the reflector, but they can also be implemented by adding optical paths into the crystal block. However, it requires the ability to measure the spatial distribution of light spread from the photosensor, which in turn increases the number and complexity of the data acquisition system. The other approach, known as the dual-ended readout method, involves attaching photosensors to both the top and bottom ends of the scintillation crystal [15,16,17,18]. The DOI is calculated by measuring the difference in the amount of light reaching each photosensor, depending on the depth of the gamma-ray interaction. While this method has a higher cost due to the need for sensors on both sides, it offers an advantage in time resolution because it can detect early direct photons that are not reflected on the crystal surface from both sides. Additionally, this method facilitates the multiplexing of sensor channel.

The dual-ended readout method has been utilized and proposed by various research groups, but its usability has significantly increased with the introduction of silicon photomultipliers (SiPMs). Unlike bulky photomultiplier tubes (PMTs), the thin SiPMs do not occupy much space even when attached to the front surface of the scintillation crystal (the surface where gamma rays enter), resulting in relatively less gamma-ray loss due to attenuation. In dual-ended readout, DOI is determined by the difference in the amount of light reaching the photosensors on the top and bottom surfaces; thus, increasing this difference enhances DOI resolution. This difference in light intensity is primarily due to the variation in travel length to reach both ends, depending on the DOI. A longer travel length increases the number of interactions with the crystal’s lateral sides, which in turn raises the probability of photon loss. For this reason, to increase photon loss associated with travel length, dual-ended readout detectors have normally used unpolished finishing on the lateral sides of the scintillation crystal, rather than polished finishing, which has better photon transmission efficiency. The surface treatment of the crystal is thus a critical factor in determining the performance of this type of detector.

Several studies have compared the DOI and TOF performance of scintillators based on surface treatment in dual-ended readout configurations [19,20,21]. These studies typically involve crystals with all lateral surfaces either polished or rough (unpolished), and they conduct these comparisons at the single crystal level rather than the crystal array level. However, extending the results from single crystals to arrays requires additional research because crystal arrays involve different light-sharing dynamics between adjacent crystals and sensors. Additionally, recent studies have reported that time resolution can be improved by modifying the conditions of only some of the lateral surfaces of the entire crystal in single-ended readout detectors [22,23]. This approach is also expected to impact both the timing resolution and DOI resolution in dual-ended readout detectors.

In this study, we propose a dual-ended block detector utilizing a dual-finishing crystal, which incorporates both unpolished and polished surfaces. For high-resolution brain-dedicated PET applications, the detector was designed using 2 mm scintillation crystals, with the block detector configured as an 8 × 8 array. The proposed technique for multiplexing SiPM signals combines strip pixels with a one-dimensional charge division method, enabling crystal identification and DOI calculation using only four signal lines, like a single-ended block detector. To compare with traditional methods, we evaluated detector blocks with all four lateral surfaces polished and with all surfaces unpolished, comparing their flood maps, energy resolution, and time resolution. The experimental results demonstrated that the proposed detector outperformed the comparison groups in terms of timing resolution. The proposed method is expected to be particularly useful in high-resolution/high-sensitivity detectors with a high aspect ratio, where extensive reflection occurs on the scintillation crystal surfaces.

## 2. Materials and Methods

### 2.1. Dual-Ended Block Detector

For the development of a high-resolution TOF detector for brain imaging application, an 8 × 8 crystal array with 2 mm pitch and 15 mm length was used for constructing block detector. This crystal array was matched with a 4 × 4 SiPM with a 4 mm pixel pitch (AFBR-S4N44P164M, Broadcom Inc., San Jose, CA, USA), so that four scintillation crystal pixels were aligned with one SiPM pixel. A 0.5 mm light guide was inserted between the scintillation crystals and the SiPM to ensure efficient light transmission.

**Figure 1:**
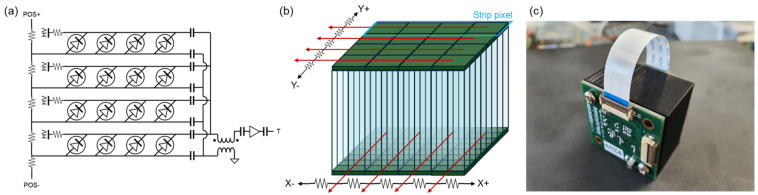
(**a**) Schematic drawing of the multiplexing circuit used in the experiment; (**b**) dual-ended detector configuration and multiplexing circuit; (**c**) photograph of the actual detector module.

To achieve efficient signal compression, a 1 × 4 pixel group from a 4 × 4 SiPM array was combined to form a strip pixel with dimensions of 4 mm × 16 mm [24]. This strip pixel was then multiplexed using a one-dimensional charge division circuit, allowing the determination of the one-dimensional interaction position through two position signals (Figure 1a). This SiPM circuit was attached to the top end of the scintillation crystal to determine the interaction position in the Y direction, and it was rotated 90 degrees and attached to the bottom end to determine the interaction position in the X direction (Figure 1b). This configuration allows the calculation of the two-dimensional interaction position within the scintillation crystal by using the four position signals from the top-end (*Y^+^* and *Y^−^*) and from the bottom-end (*X^+^* and *X^−^*) as follows:

(1) $$X=\frac{X^{+}-X^{-}}{X^{+}+X^{-}}$$

(2) $$Y=\frac{Y^{+}-Y^{-}}{Y^{+}+Y^{-}}$$

The energy was calculated by summing all position signals from both the top and bottom surfaces:

(3) $$E=X^{+}+X^{-}+Y^{+}+Y^{-}$$

The DOI index, which is used to determine the interaction depth, can be calculated using the ratio of the sensor signals from the top and bottom surfaces as follows:

(4) $$DOI=\frac{Y^{+}+Y^{-}-X^{+}-X^{-}}{X^{+}+X^{-}+Y^{+}+Y^{-}}$$

To achieve good timing resolution, high-speed timing signals were generated from both the top-end and bottom-end SiPM arrays. The signals from the four strip pixels were combined using a high-pass filter and then amplified through a high-frequency amplifier (Figure 1a).

The timestamp was determined by averaging the signals from the sensors on the top and bottom surfaces [20]:

(5) $$T={(T}_{TOP}+T_{BOT})/2$$

### 2.2. Crystal Array Configuration

**Figure 2:**
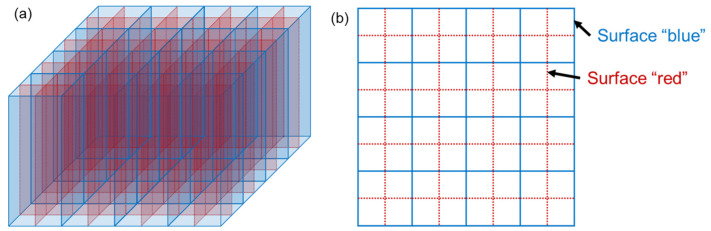
Reflector arrangement of the scintillation crystal block. (**a**) Three-dimensional view and (**b**) top view of the crystal block.

To compare the performance of the proposed dual-finishing method with the conventional approach of treating all lateral surfaces identically, three scintillation crystal blocks with the same dimensions were prepared. The dual-finishing block (DFB) was processed as follow: the surfaces marked as “blue” in Figure 2 were polished, while the surfaces marked as “red” were unpolished. Since four crystals are matched to a single SiPM pixel, this configuration allows the placement of polished surfaces at all edge regions of the SiPM pixel. Placing unpolished surfaces at the pixel’s edge (i.e., the surfaces marked as “red” were polished, while the surfaces marked as “blue” were unpolished) was not considered, as this could lead to significant light escape from the scintillation crystals, particularly those located at the edges of the crystal block, resulting in performance degradation. The comparison groups, the unpolished finishing block (UFB) and the polished finishing block (PFB), had all surfaces treated with unpolished and polished finishes, respectively. The contact surfaces with the SiPM array (top and bottom surfaces) of all scintillation crystals were polished. All the crystal blocks were fabricated using LGSO crystals from the same production lot. In all blocks, an enhanced specular reflector (ESR) was glued between the scintillation crystal pixels to ensure clear separation between the pixels. The configurations of the three blocks are summarized in Table 1.

### 2.3. Experimental Setup

Two different experimental setups were used to measure the performance of the detectors.

**Figure 3:**
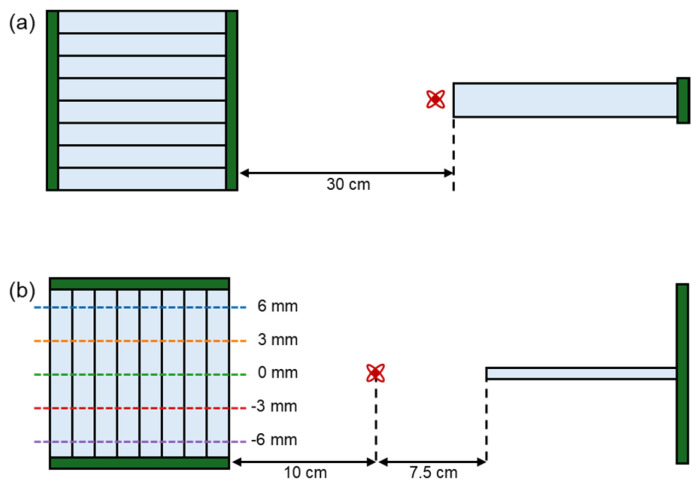
(**a**) Front-on irradiation experimental setup; (**b**) side-on irradiation experimental setup.

To acquire uniform flood maps, energy resolution, and time resolution, a front-on irradiation setup was employed, in which gamma rays were incident on the front of the scintillation crystal block (Figure 3a). For the front-on irradiation, a single pixel SiPM detector consisting of 3 × 3 × 20 mm^3^ LYSO crystal and single 4 mm SiPM (AFBR-S4N44C013, Broadcom Inc., USA) was used as a reference detector for front-on irradiation experiment. The single time resolution of the reference detector was 127.06 ps.

For measuring DOI resolution, the side-on irradiation setup was used, in which gamma rays were incident on the side of the scintillation crystal block (Figure 3b). This setup allowed for filtering gamma rays that were incident at specific depths selectively, enabling accurate DOI resolution measurements. In this experimental setup, a reference detector was constructed by coupling a slab crystal with dimensions of 16 × 0.75 × 17 mm^3^ with a 4 × 4 SiPM array (AFBR-S4N44P164M) to avoid shadow regions in the coincidence acquisition.

The position signals and timing signals from both the dual-ended detector and the reference detector were digitized using an FPGA-based DAQ system (BASP-10011, Brightonix Imaging Inc., Seoul, Republic of Korea). This DAQ system employed an 80 MHz, 12-bit ADC for energy calculation, and a tapped delay line time-to-digital convertor (TDC) was implemented in the FPGA for precise timing measurements. The TDC includes real-time bin-width calibration and has an intrinsic timing resolution of 55.6 ps FWHM [25]. A threshold voltage of 20 mV was used for the leading-edge discriminator in all experiments for timestamp pick-off. The coincidence signals were discriminated within the BASP-10011 board, with a coincidence window of 12.5 ns.

### 2.4. Data Acquisition and Analysis

In the side-on irradiation experiment, data were collected at positions −6, −3, 0, 3, and 6 mm from the center of the scintillation crystal to obtain the DOI index distribution for each crystal pixel at various depths (Figure 3b). The DOI index was converted to millimeter unit by analyzing the relationship between the central value of the DOI index distribution and the actual irradiation depth. The DOI resolution was then determined by calculating the full-width-half-maximum (FWHM) of the DOI index distribution at each interaction depth.

Energy and time resolution were calculated from data acquired through the front-on irradiation experiment. For cases where the DOI could be distinguished, the interaction depth was divided into five layers: −7.5 to −4.5 mm (DOI layer 0), −4.5 to −1.5 mm (DOI layer 1), −1.5 to 1.5 mm (DOI layer 2), 1.5 to 4.5 mm (DOI layer 3), and 4.5 to 7.5 mm (DOI layer 4). The energy resolution and timing resolution were calculated for each DOI layer, and the average of the measurements across the five layers was defined as the energy resolution and timing resolution for the respective block.

To assess the performance as a function of SiPM bias voltage, the bias voltage for the dual-ended detector was increased from 44.0 V to 48.0 V in 1.0 V increments, with data collected at each voltage level. In all cases, the analysis of timing resolution and DOI resolution was performed using only events that fell within the energy window defined by the full width at tenth maximum of the energy spectrum.

## 3. Results

### 3.1. Flood Map Quality

**Figure 4:**
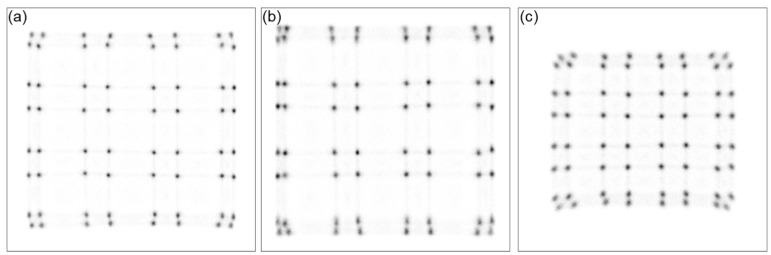
Flood map obtained from the front-on irradiation experiment. (**a**) DFB, (**b**) UFB, and (**c**) PFB.

Figure 4 shows the flood map for each block configuration. In all cases, the 64 scintillation crystals were clearly distinguishable. The size of the flood map (i.e., dynamic range) increased with the application of unpolished crystals, following the order of UFB, DFB, and PFB. However, in the UFB configuration, the spacing between the scintillation crystals located at the edges was narrower compared to the other configurations. In contrast, the PFB configuration exhibited more uniform spacing between pixels on the flood map. The proposed DFB configuration showed intermediate characteristics between UFB and PFB, and overall, it appeared to have the best quality flood map.

### 3.2. DOI Resolution

**Figure 5:**
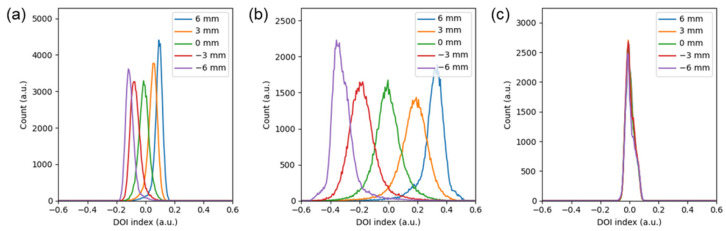
DOI index histogram of (**a**) DFB, (**b**) UFB, and (**c**) PFB.

**Figure 6:**
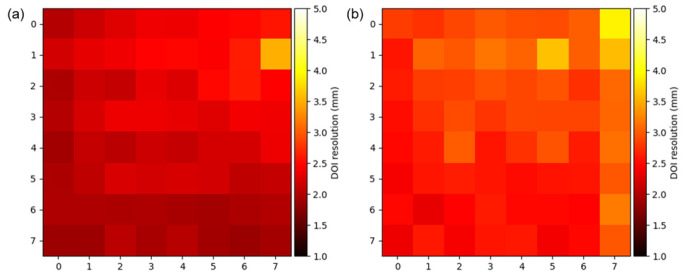
DOI resolution map for the 64 scintillation crystal pixels within the block. (**a**) DFB, (**b**) UFB.

From the side-on irradiation experiment, the DOI resolution was determined. Figure 5 shows the histogram of DOI index obtained at −6 mm, −3 mm, 0 mm, 3 mm, and 6 mm for each block. In the PFB configuration, there was no noticeable dispersion in the DOI histogram, making it impossible to measure the reaction depth. In contrast, the UFB configuration exhibited the largest dispersion in DOI index, while the DFB configuration showed less dispersion compared to UFB. The average DOI resolution across the 64 scintillation crystals was measured to be 2.21 ± 0.26 mm for UFB and 2.80 ± 0.30 mm for DFB configuration (Figure 6).

### 3.3. Energy Peak Position, Energy Resolution, and Time Resolution

**Figure 7:**
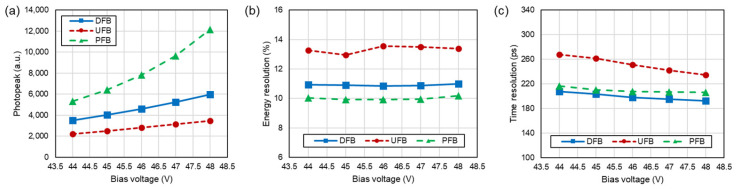
Performance variation in the detector according to bias voltage and block type. (**a**) Photopeak position, (**b**) energy resolution, and (**c**) time resolution.

Figure 7 presents the performance measurement results of each block at varying bias voltages. The results for UFB and DFB, excluding PFB where DOI could not be measured, are averaged values that account for the DOI correction, as the DOI was divided into five segments for analysis.

Figure 7a shows the photopeak position as a function of the bias voltage for each block configuration. The photopeak exhibited significant differences depending on the surface treatment conditions. In the UFB and DFB configuration, a considerable light loss was observed due to light scattering at the lateral surfaces compared to the PFB. As the SiPM bias voltage increased, the photopeak tended to rise due to the increase in photon detection efficiency and amplifying gain.

Despite the increase in bias voltage, the energy resolution remained almost constant. The PFB, which had the highest photopeak, exhibited the best energy resolution (Figure 7b). The average energy resolution at 48 V bias voltage was 10.99 ± 1.05% for DFB, 13.39 ± 1.58% for UFB, and 10.19 ± 0.55% for PFB. The particularly poor energy resolution in the UFB configuration is likely due to increased uncertainty caused by variation in the photopeak position and photon loss according to DOI.

The timing resolution improved with increasing bias voltage across all blocks, attributable to the increase in PDE (Figure 7c). While the mean-time method inherently mitigates time-walk effects by averaging the timestamps measured at the top and bottom surfaces, we further minimized the time-walk effect by calculating the time resolution for each DOI segment. Despite these efforts, the UFB, which exhibited the best DOI resolution, showed the poorest timing resolution. This indicates that in the UFB, the degradation in timing resolution due to light loss outweighed the benefits of time-walk correction. In contrast, the DFB configuration demonstrated better timing resolution than the PFB. At a bias voltage of 48 V, the timing resolution was measured at 192.0 ± 12.8 ps for DFB, 234.8 ± 17.9 ps for UFB, and 206.3 ± 9.4 ps for PFB.

### 3.4. Performance Dependency on DOI

**Figure 8:**
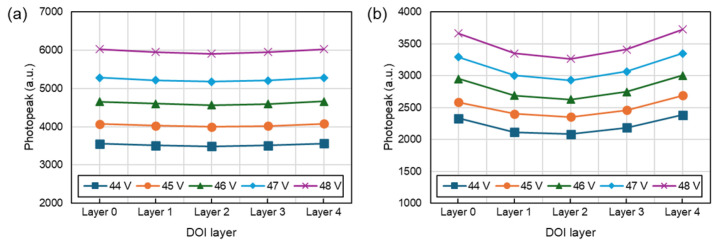
Photopeak position according to DOI layer and bias voltage. (**a**) DFB, (**b**) UFB.

We conducted a more detailed analysis of the performance variations with DOI for UFB and DFB. As shown in Figure 8, in UFB, the 511 keV photopeak at the center of the scintillation crystal (DOI layer 2) was more than 10% lower compared to the outer regions (DOI layers 0 and 4). In contrast, DFB exhibited less than a 2% difference in photopeak values between the outer and central regions, indicating relatively uniform performance regardless of depth.

**Figure 9:**
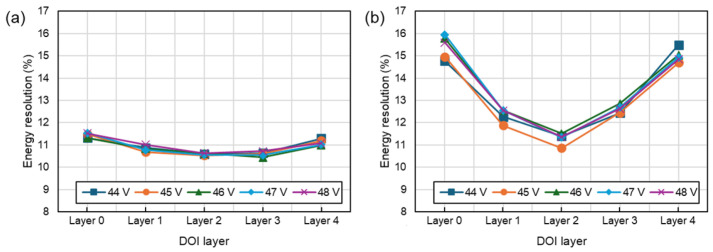
Energy resolution according to DOI layer and bias voltage. (**a**) DFB, (**b**) UFB.

Figure 9 illustrates the differences in energy resolution with respect to depth. In both UFB and DFB configuration, the energy resolution decreased at the outer regions of the scintillation crystals, where the photopeak position was higher. However, the DFB showed an energy resolution difference of less than 8%, while the UFB exhibited a difference of more than 35%. This phenomenon is likely because when the gamma ray was interacted at one end of the scintillation crystal, the light had to undergo multiple reflections to reach the sensor on the opposite end, leading to greater variation in the number of photons that reach the sensor.

**Figure 10:**
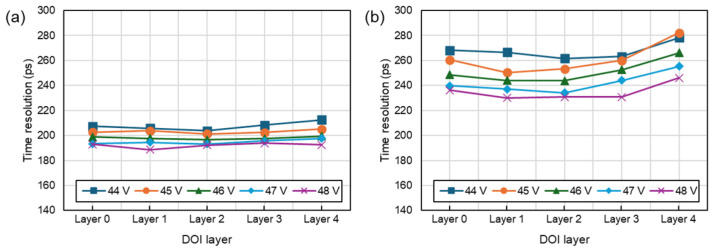
Time resolution according to DOI layer and bias voltage. (**a**) DFB, (**b**) UFB.

Figure 10 presents the variations in time resolution with respect to position. Since the mean-time method was used for timestamp calculation, the timing resolution showed minimal changes across different depths.

## 4. Discussion

In this study, we developed and evaluated the performance of a dual-ended readout detector for use brain-dedicated PET systems. Instead of the traditional all-side unpolished crystal surface treatment commonly used in dual-ended detectors, we optimized timing resolution by employing both polished and unpolished surfaces simultaneously. While the proposed method showed a slight decrease in energy resolution compared to fully polished surfaces, it still achieved a good energy resolution in the 10% range. The DOI performance also slightly decreased from 2.2 mm with the all-side unpolished treatment to 2.8 mm, but a DOI resolution below 3 mm is sufficient to compensate for the spatial resolution degradation caused by parallax errors in brain-dedicated PET systems.

The DFB demonstrated more uniform performance across different DOI layers compared to the UFB and was able to collect more optical photons. As a result, it overcame the limitations of the UFB, leading to better energy measurement performance and timing resolution.

Interestingly, when using the dual-finishing approach, although the amount of light reaching the sensor decreased compared to polished crystals, the timing resolution improved. This suggests that despite the reduced light intensity, enough early photons still reached the photosensors at both ends in DFB configuration. Moreover, the ability to measure DOI in the DFB configuration can be considered a main factor contributing to the improvement in timing resolution.

Recently, high-bandwidth oscilloscopes have become increasingly used as data acquisition systems in experimental level. Using an oscilloscope facilitates the acquisition of the full waveform of the timing signal, enabling various signal processing techniques, such as efficient dark count rejection and baseline correction, which help achieve better timing resolution. However, implementing such high-speed digitizers at the system level presents significant challenges in terms of cost and power consumption. In this study, we used an FPGA-based data acquisition system to evaluate the performance of the detector, ensuring that the measured values closely reflect the performance achievable in the system level.

The multiplexing architecture proposed in this study is advantageous because it requires only four channels for position signals. Multiplexing many SiPM signals into four channels is a common practice in PET block detectors, which means that many existing data acquisition (DAQ) systems are already optimized for this configuration. Therefore, the proposed multiplexing method offers the benefit of acquiring position signal without requiring modifications to existing DAQ systems.

While this study demonstrated that the dual-finishing approach applied to crystal blocks can improve timing resolution, there is a limitation. The conditions for light reflection/absorption at the surface are influenced not only by the crystal surface finishing but also by factors such as the type of reflector and the method of coupling the reflector and scintillator (e.g., gluing or air coupling). In this study, only the case of gluing the ESR film to the crystal using optical adhesive was considered. However, if a diffuse reflector such as barium sulfate or Toray film is used, or if an adhesive is not applied (i.e., air gap), the surface may exhibit different reflective properties. Diffuse reflectors generally offer advantages in DOI performance because they reflect light uniformly in all directions. Therefore, using a diffuse reflector might lead to better DOI resolution even with polished crystals. By further exploring these various conditions, even better performance could be achieved.

With recent improvements in the blue light detection efficiency of SiPMs, there have been numerous attempts to use Bismuth Germanate (BGO) scintillation crystals as TOF detectors. One study demonstrated that dual-ended readout can enhance the detection efficiency of Cherenkov light in BGO scintillation crystals [26]. The other study showed that by utilizing DOI information to correct for time uncertainties within the scintillation crystal, further improvements in timing resolution could be achieved [27]. This previous research only compared the performance of fully polished and fully unpolished surfaces. If the dual-finishing method proposed in this study is applied, it may lead to additional improvements in timing resolution for BGO detectors as well.

## 5. Conclusions

The proposed method exhibited slightly reduced energy resolution compared to the polished finishing method and slightly reduced DOI resolution compared to the unpolished finishing method, while achieving the best timing resolution performance. It is expected that by optimizing the performance of dual-ended detectors using this proposed method, a TOF/DOI PET system with superior image quality can be developed.

## Figures and Tables

**Table 1:** Crystal block summary.

Block Name	Surface Red	Surface Blue
DFB ^1^	Unpolished	Polished
UFB ^2^	Unpolished	Unpolished
PFB ^3^	Polished	Polished ^1^

^1^ Dual-finishing block. ^2^ Unpolished-finishing block. ^3^ Polished-finishing block.

## Data Availability

Data are contained within the article.
